# New insights in tobacco radiotoxicity: on the research of ^210^Po in modern heated tobacco product—radioactivity, distribution, and dose assessment

**DOI:** 10.1007/s11356-024-35516-8

**Published:** 2024-11-22

**Authors:** Katarzyna Szarłowicz, Sylwia Wójcik

**Affiliations:** AGH University of Krakow, Faculty of Energy and Fuels, Kraków, Poland

**Keywords:** ^210^Po, Radiological risk assessment, Alpha spectrometry, Tobacco heater, Neo cartridges

## Abstract

The purpose of the study was to assess the radiological risk to users and the environment related to the Glo system used to heat tobacco. The concentration of ^210^Po, radioactivity per cartridge, and radiation dose assessment were evaluated. For comparison purposes, to present the exposure scale, the same analyses were also performed for several traditional cigarettes. The tests were carried out on an alpha spectrometer equipped with a PIPS detector. After examining 60 samples, the concentration of ^210^Po was found in tobacco that was a filling of Neo cartridges and in traditional cigarettes. ^210^Po concentrations [in mBq∙g^−1^] in heated tobacco were higher than in unheated. After heating, a little release of polonium (mBq per cartridge) was observed. The research was also done taking into account the flavor of the Neo cartridges and cartridges from different packages. The ^210^Po concentrations in heated Neo cartridges were greater than 30 mBq∙g^−1^ and in traditional cigarettes up to 50 mBq∙g^−1^. The estimated annual effective dose for Neo cartridges was lower than that for traditional smoking. However, it cannot be concluded that the use of Neo cartridges is healthier and does not affect the environment. What is disturbing is the increase in the number of cartridges used daily, resulting in the creation of waste containing ^210^Po and, of course, a higher effective dose received by the user.

## Introduction

The polonium element was discovered by Marie Skłodowska – Curie. It has 33 known radioisotopes, of which only the ^208^Po, ^209^Po, and ^210^Po have half-lives longer than one day. ^210^Po is the most important from the environmental point of view. It occurs in the environment as a result of natural radionuclide decays in the uranium-radium series (Muggli et al. 2008; Khater 2004; Fonollosa et al. 2015; Mano et al. 2019). ^210^Po is omnipresent in the rocks and soils that make up Earth’s crust, in the atmosphere, and in the ecosystem of waters as a result of the decay of ^222^Rn and subsequent dry or wet deposition. Volcanic eruptions, fossil-fuel burning, resuspension of soil dust, and fires are other natural sources of this radionuclide (Matthews et al. 2007). ^210^Po is highly radioactive and chemically toxic. The half-life of ^210^Po is 138.4 days. Upon decay, it emits alpha radiation, which can block the epidermis but causes serious damage if it enters a living organism. The ingested ^210^Po, compared to other alpha emitters, is easily absorbed into the blood and is mainly deposited in the soft tissues of the body. Its half-life inside the body is 1 to 2 months (Ansoborlo 2014; Harrison et al. 2007).

It is normal that living organisms are constantly exposed to background ionizing radiation emitted from either natural or man-made radiation sources. However, there are some products that create a worldwide problem related to radiation exposure. Some technologically enhanced naturally occurring radioactive material (TENORM) may be found in certain consumer products. One of them is tobacco products, which are of special interest here (EPA 2023). Polonium emits alpha rays, which are carcinogenic and in particular, lead to lung cancer (Bakar et al. 2019; Jain 2021; Kamiya et al. 2015; Simon and Bouville 2015; Paretzke 1997; World Health Organization 2023a, 2023b; IAEA 2023; NEA 2023; Thomas and Symonds 2016). There are two sources of ^210^Po in tobacco. Tobacco is a plant that can uptake radioisotopes from soil or water, while particles with radioisotopes can be deposited onto plant surfaces. It should be emphasized that the large surface of the leaves and the long growth time of the plant promote the enrichment of tobacco leaves in ^210^Po (Athalye and Mistry 1972; Muggli et al. 2008). Tobacco as a plant is used in many products including pipe tobacco, cigars, cigarillos, smokeless tobacco, and herbal products for smoking. One of them is classic cigarettes, the development of which began hundreds of years ago. In cigarettes, tobacco is burnt at high temperatures. The process of smoking cigarettes involves lighting the end of a cigarette and starting a high-temperature reaction called combustion. The combustion temperature is defined as approximately 700 to 900 °C (Baker 2006, 1974). During this process, smoke is released and ash is produced, which emits more than 8000 chemical substances that are harmful or potentially harmful to health. When a cigarette is burning, polonium is volatilized, and it is known to be absorbed to submicron particles such as those found in smoke (Hoffmann and Hoffmann 1997; Zhang et al. 2021; Watson 1985).

Over the years, the composition of cigarettes has changed. Mainly, there has been a decrease of “tar,” i.e., about 3500 chemicals, including nicotine. Its contents have decreased from 38 mg of “tar” and 2.7 mg of nicotine to 12 mg and 0.95 mg over the period of 40 years (Hoffmann and Hoffmann 1997). Chemical substances found in classic cigarettes include polycyclic aromatic hydrocarbons, aromatic amines, N-nitrosamines, organic nitrates, vinylpyridine, indene, guaiacol, methylindan, and metals such as Fe, Cr, Mn, and Cu (Hoffmann and Hoffmann 1997; Zhang et al. 2021). According to research, the amount of nitrates depends on the variety of tobacco used in the production of cigarettes. The increase in nitrates is mainly associated with the burley variety of tobacco. However, the increase in nitrates has a positive effect on the combustion, reducing the occurrence of carcinogenic substances (Hoffmann and Hoffmann 1997, Cheach at al. 2018). In the 1990s, it was believed that tar substances contained 50 carcinogens, currently, there are 79 (Hoffmann and Hoffmann 1997; Yupeng and Hecht 2022).

There is a lot of research dedicated to polonium concentration in traditional cigarettes (Berthet et al. 2022; Christobher et al. 2020; Desorgher et al. 2023; Khater 2004; Kovács et al. 2014; Srivastava et al. 2018; Ghoma et al. 2023; Felix and Ntarisa 2024; Kubalek et al. 2016; Skwarzec et al. 2001), but there is not enough investigation into new tobacco cartridges with regard to heating system.

Due to the constant development of the tobacco industry in terms of modern tobacco heating systems, research was carried out in order to assess the radiological risk to users and the environment as far as the Glo heating system is concerned. The specific objectives of this work were to determine the level of the ^210^Po radionuclide in Neo cartridges but also in several samples of traditional cigarettes to demonstrate differences in the ^210^Po content in individual Neo brand cartridges available on the Polish market and to estimate the exposure of the users of Neo cartridges and Glo heater compared to the doses obtained by the smokers of traditional cigarettes. The topic was investigated due to the growing interest in modern tobacco heating systems and claims according to which they are healthy or at least healthier than traditional smoking. So far, there has been no detailed information on the radiotoxicity of these products. More and more classic cigarettes are being replaced by heated tobacco cartridges, which appeared on the Polish market in 2018. Tobacco cartridges are considered healthier because they produce less toxic and carcinogenic emissions. At the moment, despite the theory that it is a healthier form of smoking, there is no evidence that it is less harmful. The systems in question significantly reduce the temperature that affects tobacco and other compounds. In Glo system, the heating temperature is about 250 °C. Most systems use a heating blade which acts as a heater and regulates the temperature. Another way is to use the induction heating (Haziza 2016). There are many brands of heating systems on the market, most often the type of tobacco inserts is dedicated to a given heating system. For example, the IQOS heating system has dedicated Heets tobacco cartridges, and the Glo system has Neo tobacco cartridges. Tobacco cartridges, depending on the brand, differ in terms of the amount and method of packing tobacco and come in various flavors, from strong tobacco to cherry. Heated electric tobacco systems, due to their lower temperature, heat the tobacco rather than burn it. They produce no smoke or ash as a side effect. However, their product is an aerosol. According to the research, the concentration of harmful and potentially harmful substances is lower. The aerosol composition depends on the type of tobacco cartridges and its additives (Schaller et al. 2016a, b; Kaunelienė et al. 2018; Mitova et al. 2021). However, it was noticed that the amount of nicotine is comparable to that in traditional cigarettes or even lower (Meišutovič-Akhtarieva et al. 2019). In addition to harmful chemical substances, both tobacco products are radioactive. It is connected with the presence of ^210^Po radionuclide in tobacco.

## Materials and methods

**Fig. 1:**
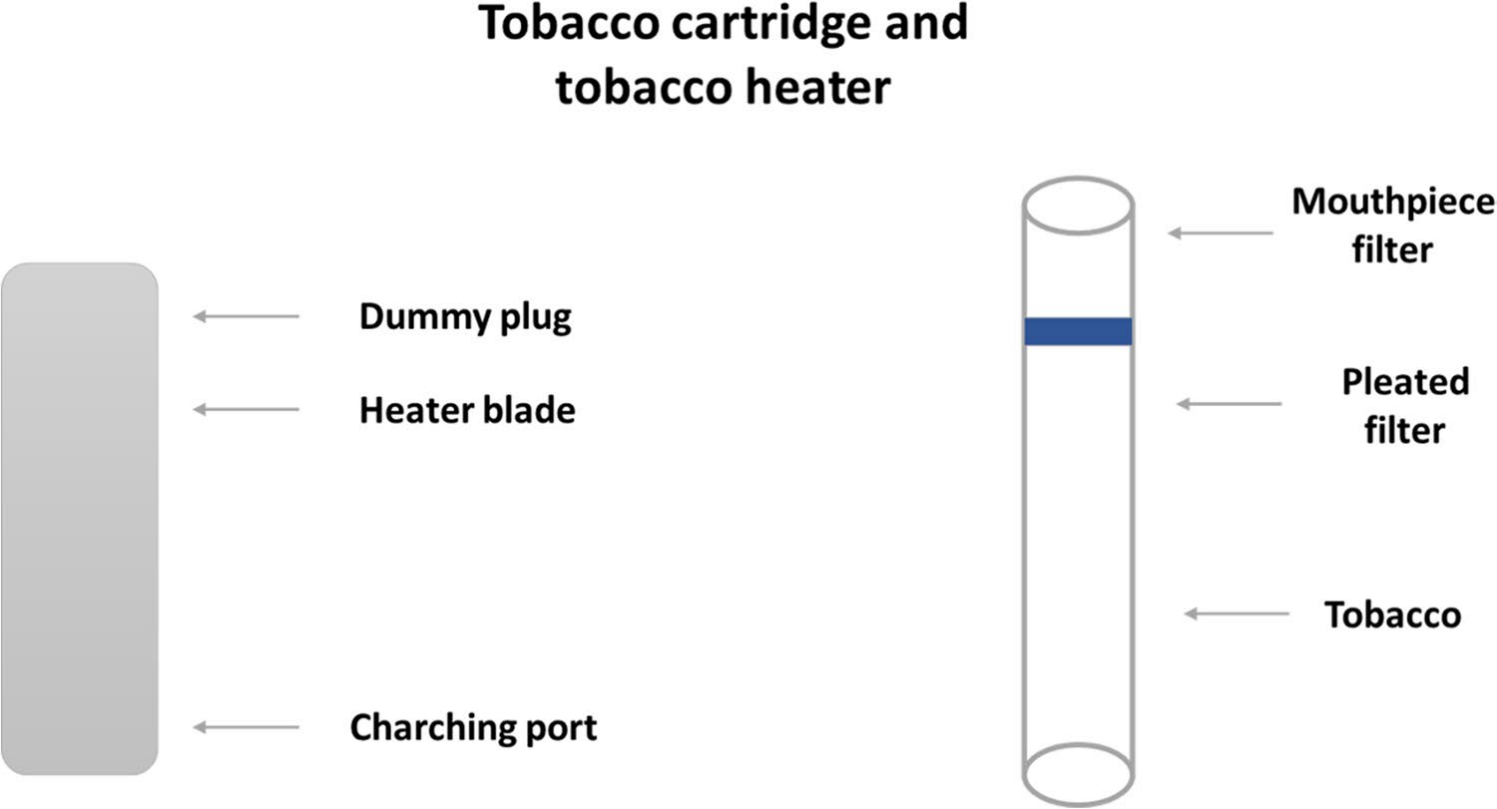
Construction of tobacco cartridge and heater

Despite a lot of data on the polonium content in traditional cigarettes, several studies of chosen traditional cigarettes and ashes were also conducted to make comparisons. The main test samples were tobacco cartridges, cigarettes, and ash obtained from users of the Glo heating system and smokers. In this study, we examined the Neo cartridges. An illustrative diagram is shown in Fig. 1.

**Fig. 2:**
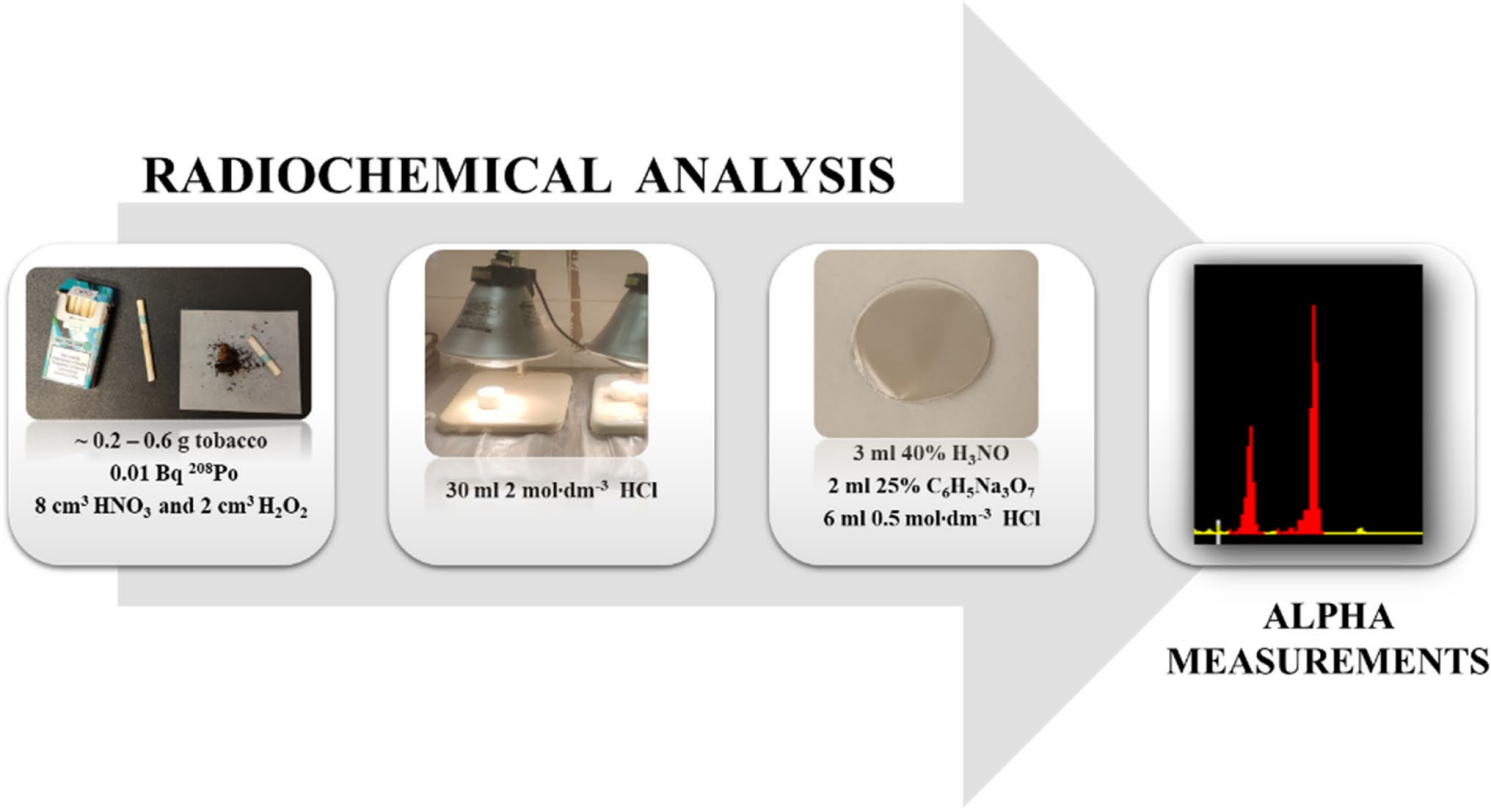
Radiochemical procedure for ^210^Po determination in tobacco samples

In all samples, polonium was determined by use of a radiochemical procedure, and measurement was done by means of alpha spectrometry. The analysis consists of a few steps which include sample preparation, microwave digestion, concentration, source preparations, and measurements. The scheme of radiochemical procedure was shown in Fig. 2. Regarding traditional smoking, ^210^Po concentrations were determined in the 5 brands of cigarettes most often bought and smoked by volunteers from the work environment. Two/three cigarettes were taken from every pack of brand. Another two/three were smoked and the ashes were gathered, separately. Due to the low tobacco content in Neo cartridges, two methods of sample preparation were used. Radioactivity was measured in single cartridges (4 per pack), and the content of two selected cartridges was mixed, which constituted one measurement sample, thus analyzing 8 cartridges from one pack.

A set of experiments were performed to optimize the parameters in the microwave digestion procedure; finally, the parameters were presented in Table 1. Only for the ash digestion was the time extended to 70 min, and the rest of the procedure steps were according to those presented in Table 1. The digestion of the samples was carried out in a closed microwave digestion system (Anton Paar); after the process, the samples were cooled to avoid loss of polonium and low recovery. All analysis (~ 0.2–0.6 g sample) was done with a tracer of ^208^Po (~ 0.01 Bq) in order to calculate the radiochemical efficiency. The analytical reagents were of high analytical grade. The samples were digested using HNO_3_ (65% Merck, high analytical purity) and H_2_O_2_ (30% Merck, suprapure) and evaporated. Then, 30 ml of 2 mol·dm^−3^ HCl was added and evaporated. Finally, the ^210^Po was deposited in the presence of 40% hydroxylamine hydrochloride solution, 25% sodium tri-citrate solution, and 0.5 mol·dm^−3^ hydrochloric acid (Fig. 2) (Flynn 1968; Szarlowicz 2019).

**Table 1:** Conditions of microwave digestion

Step	Power	Time
Power ramp	500 W	10 min
Power hold	500 W	5 min
Power ramp	1300 W	12 min
Power hold	1300 W	20 min
Cooling	0 W	-

Blank measurements were made to check the ^210^Po content in the reagents. To control the analytical procedure, the analysis with reference materials from IAEA (IAEA-447) was done. Control measurements of the concentration of polonium tracer were also made. The alpha source of ^210^Po was measured with an alpha spectrometer equipped with a PIPS detector (Canberra, model A7401). Each measurement lasted between 2 and 10 days until the accuracy of the counts was below 10%.

The radioactivity was calculated using the law of radioactive decay, and the results were converted to the same date. Measurement uncertainties were determined using the complete differential method. The radiochemical efficiency of the procedure reaches up to 98% with the majority value between 70 and 80%.

The annual effective dose was calculated by applying the dose conversion factor for adults. For the calculation of the effective dose, it has been taken (ICRP 1995) that the lung absorption of ^210^Po by inhalation of cigarette smoke is of the moderate (M) type.

$$E=C\cdot f\cdot n\cdot t$$

where *E* is the annual effective dose; *C* is the radioactivity of the intake of ^210^Po [mBq/cartridge/cigarettes] calculated as the differences between the radioactivity of unheated and heated cartridges and unburned and burned cigarettes; *f* is the dose conversion factor 3.3 μSv Bq^−1^; *n* is the number of cigarettes smoked (20 per day); *t* is the duration of smoking (365 days).

## Results

**Fig. 3:**
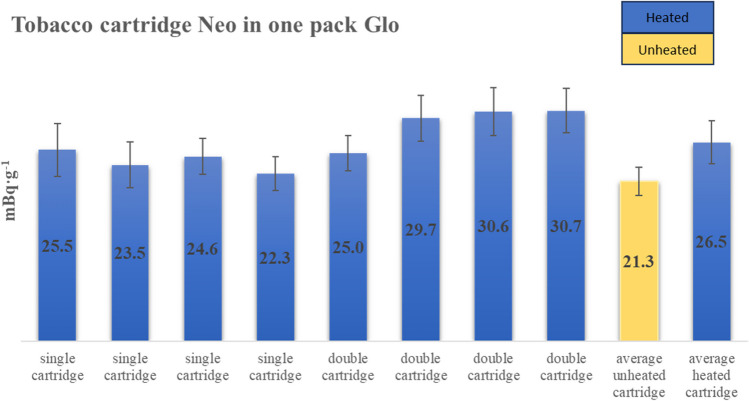
The concentration of ^210^Po in chosen cartridges from one Glo pack

Totally, 60 samples were analyzed in terms of ^210^Po radioactivity. The results present the radioactivity of ^210^Po in cartridges taken from users, as well as in cartridges depending on taste and for cartridges from one package. Also, several brands of commonly accessible cigarettes were examined, and the radioactivity of tobacco and ash was shown. In order to check the distribution of ^210^Po concentration in pack (20 cartridges), research was carried out. In Fig. 3, the results for almost all Neo cartridges (16 cartridges—8 single, 4 double) taken from one pack were shown. The average concentration in unheated tobacco from the same packet of Neo equals 21.3 ± 1.8 mBq∙g^−1^, as well as for heated it was 26.5 ± 2.85 mBq·g^−1^.

**Fig. 4:**
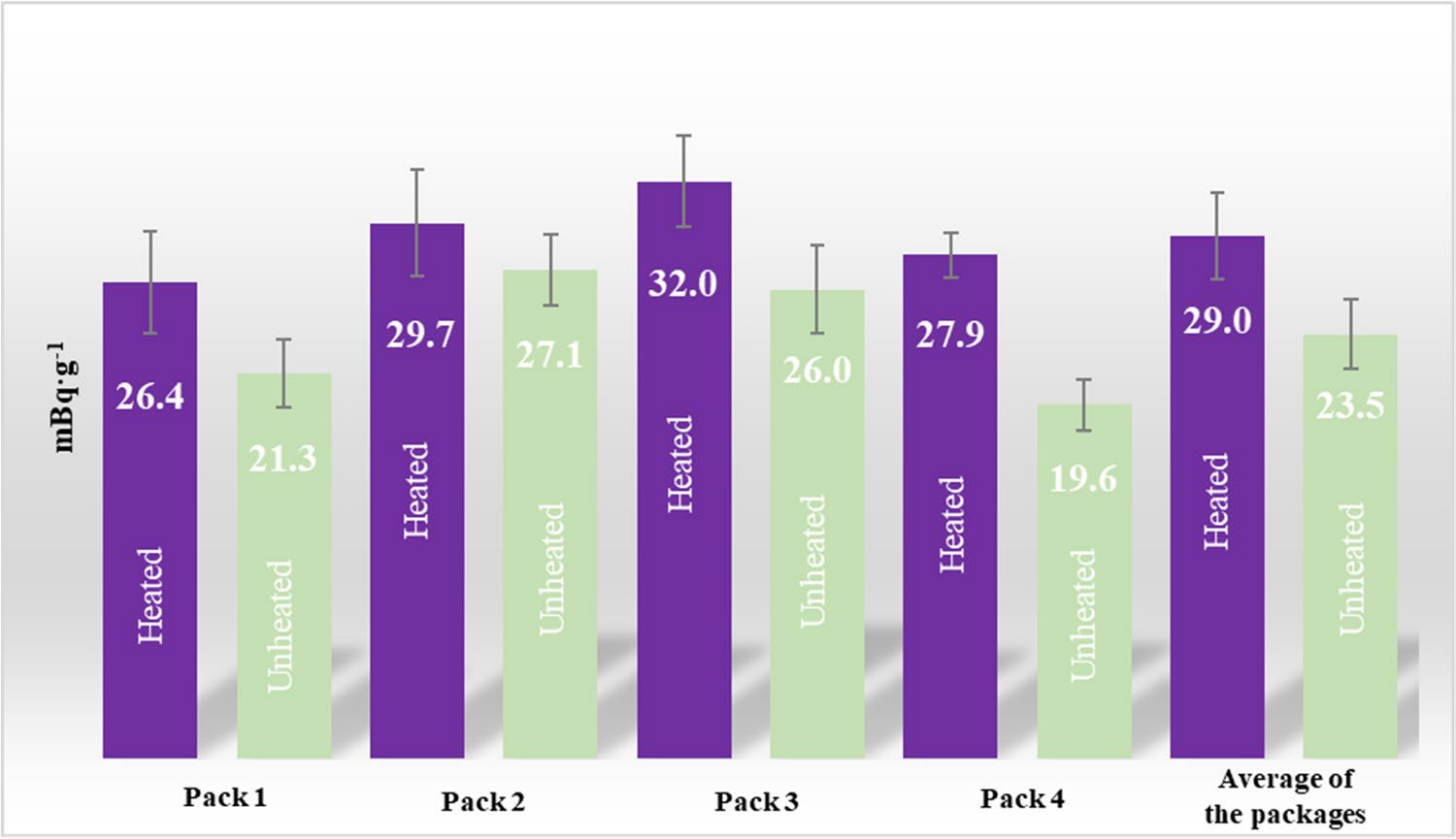
The average concentration of ^210^Po in chosen cartridges in a randomly chosen Glo pack

In Fig. 4, the average value of ^210^Po concentration was presented in cartridges taken from four packets of Neo mint. The average concentration of ^210^Po from all packs is 29.0 ± 2.4 mBq∙g^−1^ for heated and 23.5 ± 1.9 mBq·g^−1^ for unheated. The results obtained for individual packages do not differ significantly from the average values.

**Fig. 5:**
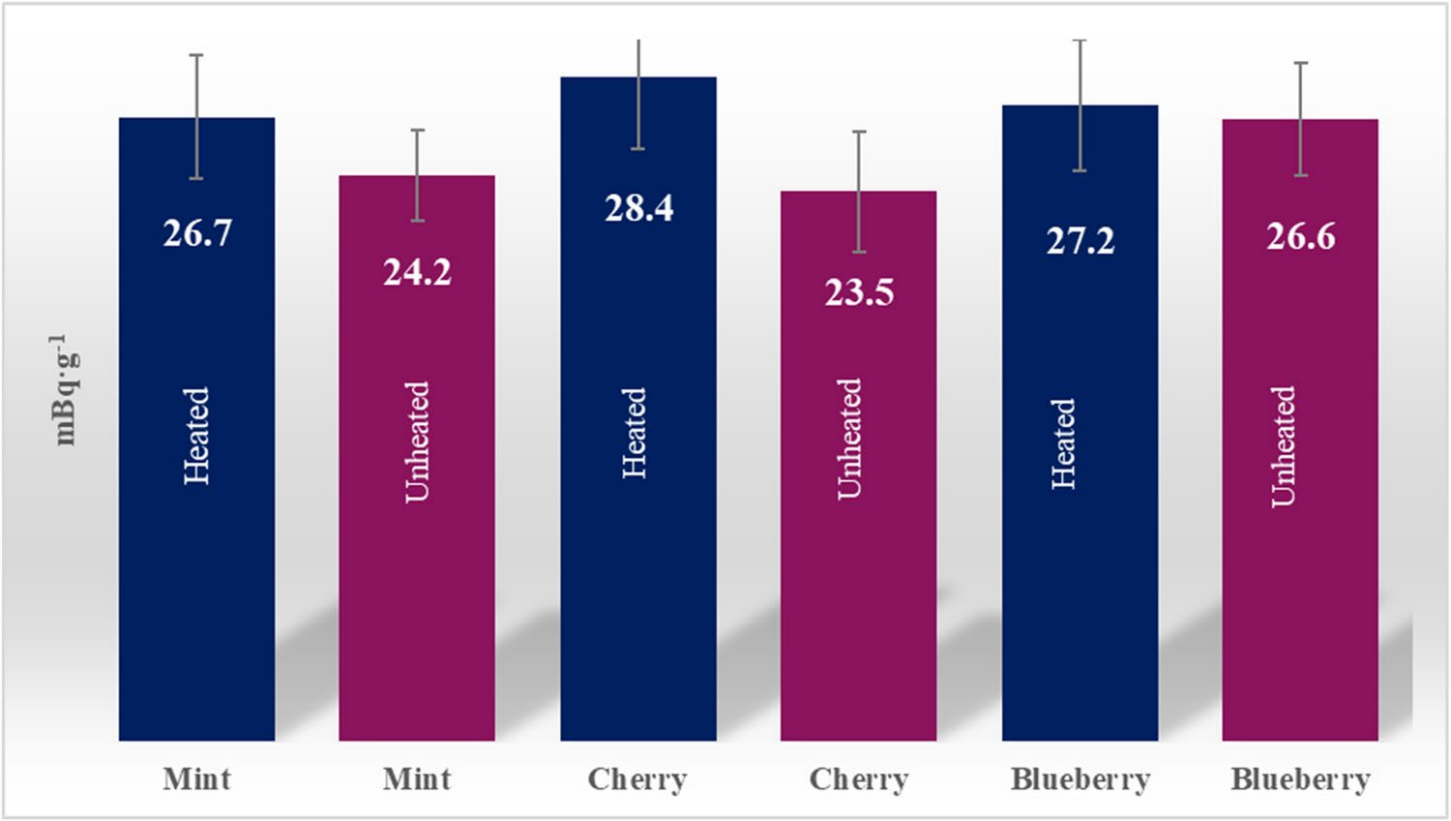
The average concentration of ^210^Po in flavor cartridges

In Fig. 5, the comparison of the average level of ^210^Po concentration in different packages (four mint, three cherry, and three blubbery) was shown in dependence of flavor. In addition, the average concentration values for heated and unheated samples were taken into account. In all cases, the average concentration of ^210^Po was below 30 mBq∙g^−1^ for heated and from 23.5 to 26.6 mBq∙g^−1^ for unheated and was lower than in heated.

Regarding traditional cigarettes, all the results of the average concentration of ^210^Po were presented in Table 2. The value was in the range of 20.6 ± 2.7 mBq∙g^−1^ and 49.4 ± 5.6 mBq∙g^−1^. The highest concentration of ^210^Po was determined in the sample of LM Link Blue, whereas the lowest concentration was determined in Marlboro Gold. In the ashes, the concentration of ^210^Po was between 18.1 ± 1.5 mBq∙g^−1^ and 27.2 ± 2.7 mBq∙g^−1^ (Table 2).

**Table 2:** Concentration of ^210^Po in traditional cigarettes (C) and ash (A)

Sample	Brand/ash	^210^Po [mBq∙g^−1^]	± [mBq∙g^−1^]
C1	LM Link Blue	49.4	5.6
A1	LM Link Blue	27.2	2.6
C2	LM Forward	25.0	4.8
A2	LM Forward	22.0	2.0
C3	Marlboro Ice	26.5	3.8
A3	Marlboro Ice	18.1	1.5
C4	LM Blue	27.7	3.0
A4	LM Blue	20.4	2.4
C5	Marlboro Gold	20.6	2.7
A5	Marlboro Gold	18.8	1.4

### Estimation of the annual effective dose

In Tables 2 and 3, the radioactivity of ^210^Po per cigarette and annual radiation dose rate were shown. The calculations were made for traditional cigarettes and Neo cartridges.

**Table 3:** Average radioactivity of ^210^Po and effective dose for cigarettes and Neo cartridges

Brand	mBq/cigarette/Neo cartridge	μSv/year
LM Link Blue	16.64	151
LM Forward	13.77	126
Marlboro Ice	14.58	142
LM Blue	15.51	150
Marlboro Gold	11.35	102
Neo Blueberry	8.76	4.3–40
Neo Mint	6.83	7.9–48
Neo Cherry	8.96	22–67
Mean cigarettes	14.37	134
Mean Neo	8.18	26.8

## Discussion

The results obtained indicate that there is little difference in the amount of ^210^Po in the packet of Neo cartridges. In every heated sample, the concentration of ^210^Po radionuclide was higher than in the unheated samples. As a result of concentration, the radioactivity of ^210^Po specified in mBq·g^−1^ in cartridges increases, which is caused by heating tobacco. This increase in concentration is likely related to the low heating temperature of the tobacco and the localized, short-term heating, which reduces the tobacco mass. Due to the short duration and relatively low temperature of heating, the losses caused by volatility are not as significant as in traditional smoking. Therefore, while the total amount of ^210^Po (in mBq per cartridge) is lower after heating, the concentration of ^210^Po (in mBq·g^−1^) is slightly higher.

The diversity of the concentration appears to be random. Across several packets of cartridges, one can find packetcontaining either lower or higher levels of ^210^Po. This is observed in both heated and unheated samples. However, the difference in concentration is only a few percent.

Taking into account cartridges with the same flavor from different packages, a slight diversity in the ^210^Po content was also observed. The tendency of lower ^210^Po concentration in unheated cartridges is maintained. This observation also holds true for cartridges with different flavors.

It seems that these small differences in the results are not due to flavor additives but are the result of the use of tobacco mixtures or tobacco with noticeable differences in tobacco radioactivity. In the analyzed cartridges purchased in Polish stores, the recorded polonium concentration levels are at a similar level. In the current scientific literature on tobacco heating systems, there are only a few studies in which authors have attempted to address the issues related to radiological exposure. These results are in line with those obtained by (Berthet et al. 2022). They calculated the radioactivity of ^210^Po in IQOS Heets bronze label. The level was 6.9 mBq/cartridge, in our research was at the same level or higher. Generally, it can be said that obtained ^210^Po concentrations in fresh tobacco are within the limits observed in conventional cigarettes and tobacco cartridges.

Similarly to traditional cigarettes, in modern tobacco cartridges, the source of polonium is the tobacco that constitutes their filling. The amount of ^210^Po in tobacco depends on the type of tobacco. In particular, it depends on the location of tobacco cultivation. On the one hand, tobacco absorbs radionuclides, including polonium, through the surface system, and on the other, due to the huge sorption surface of tobacco leaves. It contains ^210^Po from the air originating from decays of ^222^Rn. When comparing the radioactivity of ^210^Po in cartridges used in modern tobacco heating systems with the radioactivity obtained for traditional cigarettes, the amount of tobacco for these tobacco products should be taken into account. The radioactivity of polonium (in Bq) may be lower in tobacco cartridges as a result of the lower amount of tobacco used. Here, the type and country of origin of tobacco are an important factor. In countries with higher background radiation, growing tobacco plants will have higher concentrations of this radionuclide. An important issue is whether, due to the lower radioactivity of the ^210^Po contained in the cartridges, less polonium enters the smoker’s body.

An attempt was also made to estimate radiological risk. The radiation dose from radioisotopes (^210^Po and ^210^Pb) for classic cigarettes is 158 µSv per year (Boryło et al. 2022). In our studies, the annual effective dose for traditional smoking was 134.62 µSv per year. In studies conducted in India, the concentration of radionuclides for cigarettes ranged from 68.6 to 132.8 Bq·year^−1^ for ^210^Po. The effective annual dose was 153.4 µSv/year for ^210^Po. For other countries, the doses differed. For Greece—124 µSv/year, for Slovakia—61 µSv/year, for Hungary—185.6 µSv/year, and even 223 µSv/year for Vietnam. While examining ^210^Po concentrations in many countries, Vietnam had a great advantage over the rest, with an average of 29 mBq per cigarette (Berthet et al. 2022; Desorgher et al. 2023). Poland in this ranking had an average concentration of ^210^Po in one cigarette of 13 mBq. In the conducted research, the average radioactivity of ^210^Po in 1 cigarette was 14.37 mBq. High effective doses of traditional cigarettes were recorded in India, where the dose ranged from 409.8 to 691.6 μSv. These doses are even twice as high as those for Poland (Christobher et al. 2020; Kovács et al. 2014; Srivastava et al. 2018). Taking into account the estimated dose of ^210^Po from Neo cartridges, it can be observed that it is lower than that from traditional smoking. Two methods of calculation were taken into account, one based on the average concentration of ^210^Po in a pack, and the second based on a single cartridge, directly measuring the ^210^Po content in the cartridge and in a randomly selected unheated cartridge. The estimated annual dose was in the range from ~ 5 to ~ 60 µSv/year (Table 3). The values are overestimated. It was assumed that the resulting aerosol would completely enter the lungs. In fact, the aerosol is not completely inhaled but remains in the users’ surroundings, so it enters the environment. The calculated effective dose radiation was much lower than that of traditional cigarettes.

It should be remembered that tobacco for both classic cigarettes and tobacco cartridges comes from different parts of the world. And, of course, the dose is related to the concentration of polonium in the tobacco that fills the products. Based on the research, it can be assumed that there is less radiological contamination, but this cannot be stated definitively due to the growing popularity of modern heating systems, or more specifically, the increasing number of devices being used. Analyses and statistics predict an upward trend in the popularity of tobacco products, as young people are the most likely to use them or e-cigarettes. Currently, smokeless tobacco products are under increased scrutiny due to previous studies that have shown their harmful effects (World Health Organization 2023a, 2023b; Chenga et al. 2023; Statista 2023; Chugh et al. 2023; CSO 2019; Bekki et al. 2021). According to the Central Statistical Office (CSO), cigarette and tobacco production is increasing every year. Smoking rates among adults from 2009 to 2019 have remained at a similar level. People in their 50 s are the most likely to report smoking tobacco products. On average, one person smokes 1.5 packs of cigarettes or other tobacco products per day (Statista 2021; Atlas Big 2023).

## Conclusions

Based on the obtained results, the following can be concluded:

1. The proposed way of determining the tobacco sample is convenient and is described with high efficiency.
2. The ^210^Po concentration in tobacco cartridges is lower than or comparable to the concentration in traditional cigarettes and depends on the mixture of tobacco that constitutes the filling.
3. The tested tobacco cartridges had a lower ^210^Po content (in Bq) than traditional ones.
4. Primary studies indicate that effective dose radiation from heaters Neo cartridges is lower than calculated from traditional smoking.
5. A tobacco cartridge used is waste that contains radioactivity, which can increase the dose of radiation in the environment.

In summary, the radiation dose is used to estimate the health effects associated with exposure to ionizing radiation received from tobacco products. On the basis of the results obtained, it can probably be concluded that the dose level received by people when using the Glo system and Neo cartridges is lower than in the case of the dose received during traditional smoking. Nevertheless, it should be emphasized that the dose level will depend primarily on the number of cartridges used. Unfortunately, it is becoming more and more common for people to smoke tobacco cartridges during the day, so if the number of cartridges smoked is large, the dose automatically obtained will be higher.

It is not possible to clearly determine whether tobacco cartridges are radiologically safer, due to the multitude of factors that influence the radiation dose received by the user. Therefore, it is necessary to investigate various modern systems to heat tobacco cartridges in order to illustrate the problem of radiation exposure.

There are too many factors that can influence the radiological exposure of a standard user. The method and time of inhalation, the time of heating the tobacco in the heater, the amount of smoke produced that remains in the smoker's environment, the number of refills used per day, the type of heater, and the type of refills are undoubtedly important here.

The topic is extensive and problematic and worth continuing. To systematize knowledge, inform the general population, and assess exposure to TENORM in the environment.

## Data Availability

All data analyzed during this study are included in this published article. Additional data is available on request.
